# A Rare Case of Xeroderma Pigmentosum: Nivolumab Treatment for Three Cutaneous Malignancies with Clinical and Metabolic Imaging Correlation

**DOI:** 10.3390/diagnostics15080979

**Published:** 2025-04-12

**Authors:** Ilaria Proietti, Riccardo Pirisino, Giulia Azzella, Vincenzo Coppolelli, Maria Elisabetta Greco, Emanuele Casciani, Concetta Potenza, Luca Filippi

**Affiliations:** 1 Dermatology Unit “Daniele Innocenzi”, “A. Fiorini” Hospital, Via Firenze, 1, 04019 Terracina, Italy; ilaria.proietti@uniroma1.it (I.P.); azzellagiulia1996@gmail.com (G.A.); vincenzo.coppolelli@uniroma1.it (V.C.); mariaelisabetta.greco@uniroma1.it (M.E.G.); concetta.potenza@uniroma1.it (C.P.); 2 Department of Nuclear Medicine, Santa Maria Goretti Hospital, AUSL Latina, 04100 Latina, Italy; r.pirisino@ausl.latina.it; 3 Department of Biomedicine and Prevention, University of Rome “Tor Vergata”, Via Montpellier 1, 00133 Rome, Italy; emanuele.casciani@uniroma2.it

**Keywords:** metabolic imaging, PET/CT, metastatic melanoma, immunotherapy, precision medicine

## Abstract

Xeroderma pigmentosum (XP) is a rare autosomal recessive disorder characterized by extreme ultraviolet (UV) sensitivity, predisposing patients to multiple cutaneous malignancies. We present the case of a 26-year-old male with XP diagnosed with three distinct skin cancers: superficial spreading melanoma (SSM), basal cell carcinoma (BCC), and squamous cell carcinoma (SCC). Among these, the melanoma had metastasized. A computed tomography (CT) scan revealed a suspicious pulmonary nodule, prompting further metabolic characterization via positron emission tomography/computed tomography (PET/CT) with ^18^F-fluorodeoxyglucose ([^18^F]FDG). The scan detected significant hypermetabolism not only in the lung lesion but also in an unsuspected right parotid gland lesion, refining disease staging and guiding treatment decisions. The patient underwent immunotherapy with nivolumab, achieving a complete metabolic response in both metastatic lesions, as confirmed by follow-up PET/CT. This case underscores the critical role of [^18^F]FDG PET/CT in staging and treatment monitoring for selected patients with XP, a population in which advanced imaging is rarely employed. Moreover, the patient’s remarkable response to immunotherapy suggests a potential link between XP-related DNA repair defects and increased sensitivity to PD-1 blockade. These findings highlight the importance of integrating metabolic imaging into XP management and warrant further investigation into the immunogenicity of XP-associated malignancies.

**Figure 1:**
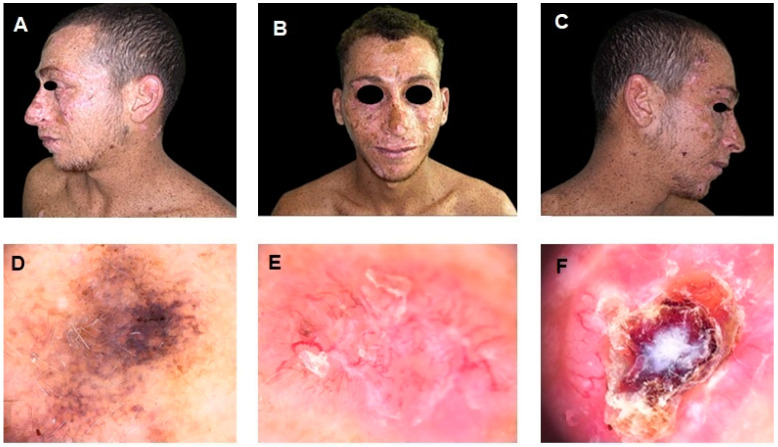
(**A**–**C**) Clinical images of a 26-year-old Egyptian male with a history of xeroderma pigmentosum (XP), a rare autosomal recessive disorder characterized by extreme sensitivity to ultraviolet (UV) radiation, leading to early-onset cutaneous malignancies. The patient exhibits multiple pigmented lesions, extensive freckling, and actinic damage, particularly in sun-exposed areas. (**D**–**F**) Dermoscopic images of different skin malignancies. Image (**D**) shows a superficial spreading melanoma (SSM), displaying an irregular pigment network, asymmetry, multiple colors, and regression structures. Image (**E**) depicts a basal cell carcinoma (BCC), characterized by arborizing vessels, a pink background, and superficial scaling. Image (**F**) illustrates a squamous cell carcinoma (SCC), presenting a central keratinous core, ulceration, and peripheral erythema, indicative of an advanced lesion. The SSM was surgically excised and histologically confirmed. Both the BSC and SCC were classified as unresectable following a multidisciplinary evaluation, in accordance with current clinical criteria. As supported by the literature and international guidelines, unresectability was defined by factors such as the extent of local invasion, tumor location near critical structures (e.g., orbit or skull base), and the high risk of significant functional or cosmetic morbidity associated with surgery [1,2]. Given the complexity and rarity of the patient’s clinical condition, further diagnostic examinations were conducted, including ultrasound of the head and neck lymph nodes, both of which were negative for metastatic recurrence. However, a chest computed tomography (CT) scan revealed a nodular formation with a maximum diameter of 16 mm in the lower lobe of the left lung, suspected to be a metastatic lesion. To achieve a metabolic characterization of the lung nodule, a positron emission tomography/computed tomography (PET/CT) scan with ^18^F-fluorodeoxyglucose ([^18^F]FDG) was requested.

**Figure 2:**
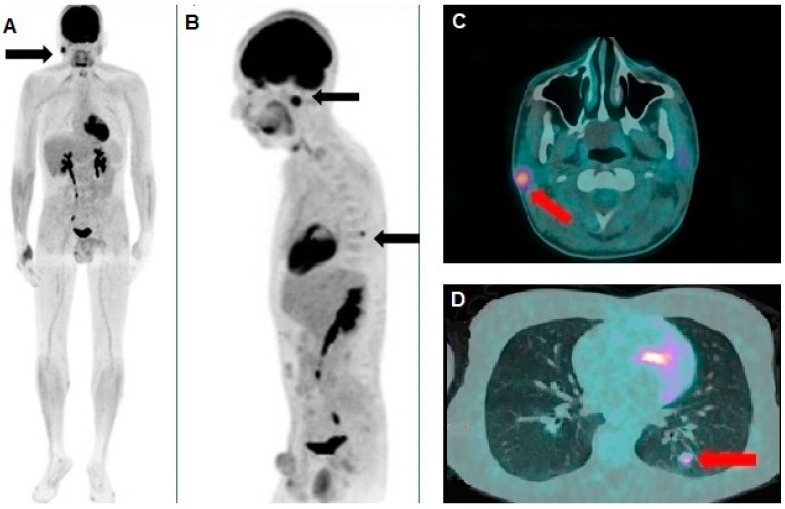
(**A**) Whole-body [^18^F]FDG PET/CT revealed significant hypermetabolism in the right parotid gland, as indicated by the black arrow, suggesting a metabolically active lesion. (**B**) Oblique sagittal reconstruction showing pathological [^18^F]FDG uptake both in the parotid gland and in an additional focal area in the left hemithorax, compatible with a pulmonary nodule (black arrows). PET images were assessed using both qualitative and quantitative analyses. Specifically, any lesion demonstrating tracer uptake higher than the surrounding background and not attributable to physiological activity was considered potentially pathological. Using dedicated software (PET VCAR, version 4.7, GE Healthcare, WI, USA), the maximum standardized uptake value (SUVmax) was calculated. Additionally, each lesion was segmented by applying a threshold of 42% of the SUVmax, and the metabolic tumor volume was automatically measured. The axial PET/CT fusion images of the skull (**C**) provided further characterization of the lesions, showing hypermetabolism in a small nodular formation within the right parotid gland (red arrow), with a maximum standardized uptake value (SUVmax) of 6.9 and a metabolic tumor volume (MTV) of approximately 3 cubic centimeters (cm^3^). Additionally, (**D**) the known pulmonary nodule in the apical segment of the left lower lobe (red arrow), previously identified on chest CT, exhibited an SUVmax of 4.8 and an MTV of about 0.5 cm^3^. Considering the patient’s clinical history, both lesions were interpreted as metastatic disease from the XP-associated melanoma. Consequently, the patient started anti-programmed cell death protein 1 (anti-PD-1) therapy with nivolumab at a dose of 480 mg every four weeks, which was well tolerated without significant adverse effects.

**Figure 3:**
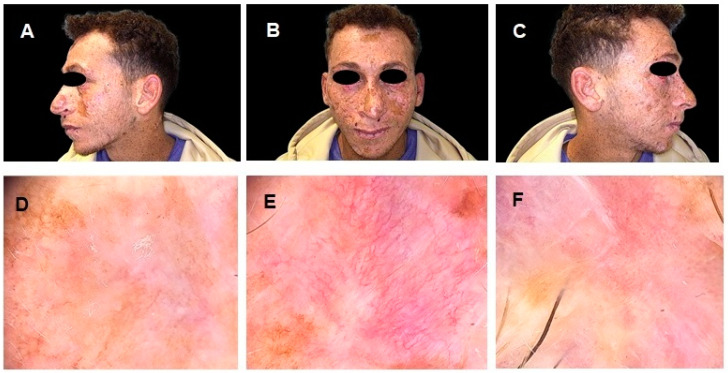
Three months after the start of therapy, the patient was clinically re-evaluated. (**A**–**C**) The physical examination showed a significant improvement in previously observed skin lesions, with no signs of relapse of the previously excised SSM (**D**) and a marked regression, under dermoscopic examination, of both the BSC (**E**) and the SCC lesions (**F**). To assess the response to systemic therapy, the patient also underwent a follow-up PET/CT scan.

**Figure 4:**
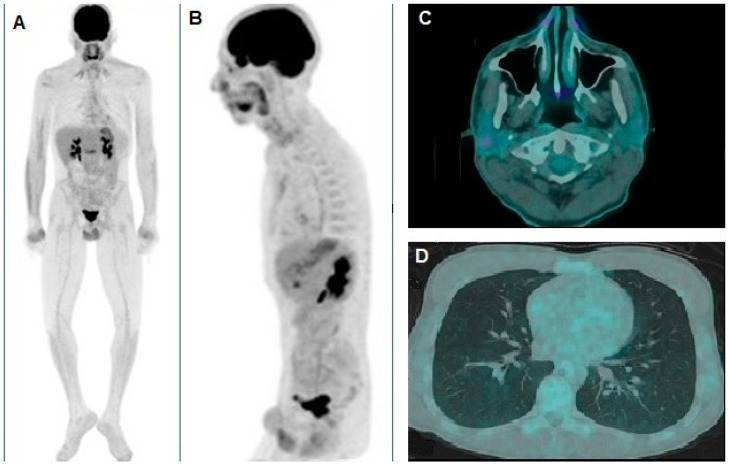
(**A**) Whole-body and (**B**) oblique sagittal [^18^F]FDG PET/CT demonstrated complete resolution of the pathological foci of tracer uptake in the thorax and the right parotid gland, as documented by the pre-treatment scan. For the qualitative and quantitative evaluation of the images, the approach described above was followed. The axial PET/CT fusion images also clearly depict the corresponding complete disappearance of the nodules in the right parotid gland (**C**) and the left lung (**D**). The patient, after 6 months of follow-up, is still in complete remission, under immunotherapy treatment, with no signs or symptoms of immunotherapy-related toxicity. Xeroderma pigmentosum (XP) is a rare autosomal recessive genetic disorder characterized by extreme sensitivity to UV light [3]. The underlying defect involves germline biallelic loss-of-function mutations in genes of the nucleotide excision repair (NER) pathway, leading to an accumulation of unrepaired DNA damage [4,5,6,7]. Consequently, patients with XP are highly predisposed to developing multiple cutaneous malignancies of different histological types [8,9]. Programmed cell death protein 1 (PD-1) is an immune checkpoint receptor expressed on lymphocytes, and its ligands, PD-L1 and PD-L2, inhibit immune activation, promoting self-tolerance [10,11]. Tumor-infiltrating lymphocytes often exhibit high levels of PD-1, while cancer cells and elements of the tumor microenvironment frequently express PD-L1. This mechanism enables tumors to evade immune surveillance by suppressing T-cell activity. Given this, immune checkpoint inhibitors targeting PD-1/PD-L1 have emerged as an effective therapeutic strategy, restoring T-cell activation and triggering an anti-tumor immune response. Moreover, studies have demonstrated a correlation between response to checkpoint blockade therapy and tumor mutational burden (TMB) [12,13]. Since patients with XP exhibit a severe DNA repair deficiency, it is hypothesized that their tumors harbor an elevated mutational load, potentially making them more responsive to immune checkpoint blockade. Mechanistically, the extreme sensitivity of XP-related tumors to PD-1 blockade may be linked to the defective NER pathway, which results in a distinctive mutational landscape. Patients with XP accumulate UV-induced DNA damage that is not effectively repaired, leading to a high TMB and enrichment of UV-signature mutations (notably C > T transitions). These mutations can generate a large number of neoantigens, enhancing tumor immunogenicity [14]. Furthermore, recent studies suggest that NER deficiency may also promote a pro-inflammatory tumor microenvironment, characterized by increased interferon signaling and antigen presentation machinery, which may further sensitize tumors to immune checkpoint inhibition [15]. In this case, we administered nivolumab to a patient with metastatic localizations under the assumption that immune checkpoint inhibitors could enhance endogenous immune responses and that the high mutational burden associated with XP might be a predictive factor for response. Consistently, the patient demonstrated a remarkable therapeutic outcome. Notably, [^18^F]FDG PET/CT imaging has rarely been utilized in patients with XP, although its application in metastatic melanoma is increasing [16,17,18,19]. In our case, [^18^F]FDG PET/CT played a crucial role in both staging and treatment monitoring. [^18^F]FDG PET/CT was chosen due to its superior sensitivity in detecting early metabolic changes associated with malignancies, which often precede anatomical alterations observable on CT or MRI [20]. This modality allows for the identification of both overt and occult lesions, thereby enhancing staging accuracy. In this case, the ability of PET/CT to uncover an unexpected hypermetabolic focus in the right parotid gland significantly influenced clinical decision-making, underlining its advantage in complex cases such as those encountered in patients with XP. The decision to perform [^18^F]FDG PET/CT, although not standard in XP management, was justified by indeterminate thoracic findings on CT. Pre-treatment PET/CT facilitated a more precise staging by identifying a previously unrecognized hypermetabolic lesion in the parotid gland, refining the assessment of disease burden. In the present case, the parotid lesion was interpreted as a metastasis from the known melanoma, primarily based on the natural history of melanoma, which is associated with a higher propensity for metastasis compared to squamous cell carcinoma. While no distinct clinical features allow for a definitive differentiation between a melanoma metastasis and one arising from squamous cell carcinoma, the overall clinical context and the pattern observed on [^18^F]FDG PET/CT render a melanoma origin more likely. Although histopathological confirmation would be ideal for a conclusive diagnosis, the robust response to immunotherapy further supports this interpretation. Additionally, PET/CT proved instrumental in evaluating the patient’s response to immunotherapy, demonstrating complete metabolic resolution of both the parotid and pulmonary lesions. In this respect, PET-based quantitative parameters (i.e., SUVmax and MTV) were key indicators of active disease, prompting the initiation of immunotherapy and providing an objective assessment of overall tumor burden. Follow-up imaging demonstrated complete metabolic resolution, as evidenced by the normalization of these values, thus confirming the effectiveness of the therapeutic intervention [21]. This highlights the potential utility of [^18^F]FDG PET/CT in patients with XP with ambiguous radiological findings, particularly in guiding therapeutic decisions and monitoring treatment efficacy. In conclusion, this patient with XP showed a remarkable response to nivolumab, reinforcing the potential impact of checkpoint inhibitors in this unique genetic context. We propose that the DNA repair deficiency in XP may lead to a heightened mutational load, enhancing tumor immunogenicity and responsiveness to PD-1 blockade. To validate this hypothesis, further studies should evaluate the immune landscape and mutational burden of XP-associated tumors. Additionally, identifying recurrent mutational patterns in different XP-related skin malignancies may provide insights into factors influencing sensitivity or resistance to nivolumab. In this regard, it should be noted that this study is based on a single-case report, which limits the generalizability of the findings. Case studies are inherently subject to selection bias and may not capture the full spectrum of clinical variability seen in larger patient populations. Therefore, while the observed response to immunotherapy and the role of [^18^F]FDG PET/CT in refining staging are compelling, these results should be interpreted with caution. Future studies involving larger cohorts are necessary to validate these observations and further elucidate the clinical utility of this imaging modality in managing XP-associated malignancies. Despite these limitations, our experience underscores the need for additional research into optimal therapeutic strategies for XP-associated malignancies. While immune checkpoint inhibition appears promising, integrating advanced imaging modalities such as [^18^F]FDG PET/CT into routine clinical practice may enhance disease assessment and ultimately improve patient management and outcomes.

## Data Availability

The original contributions presented in this study are included in the article. Further inquiries can be directed to the corresponding author.

## References

[1] Stratigos A.J., Garbe C., Dessinioti C., Lebbe C., Bataille V., Bastholt L., Dreno B., Fargnoli M.C., Forsea A.M., Frenard C. (2020). European Interdisciplinary Guideline on Invasive Squamous Cell Carcinoma of the Skin: Part 1. Epidemiology, Diagnostics and Prevention. Eur. J. Cancer.

[2] Schmults C.D., Blitzblau R., Aasi S.Z., Alam M., Andersen J.S., Baumann B.C., Bordeaux J., Chen P.-L., Chin R., Contreras C.M. (2021). NCCN Guidelines^®^ Insights: Squamous Cell Skin Cancer, Version 1.2022. J. Natl. Compr. Cancer Netw..

[3] Black J.O. (2016). Xeroderma Pigmentosum. Head Neck Pathol..

[4] Kleijer W.J., Laugel V., Berneburg M., Nardo T., Fawcett H., Gratchev A., Jaspers N.G.J., Sarasin A., Stefanini M., Lehmann A.R. (2008). Incidence of DNA Repair Deficiency Disorders in Western Europe: Xeroderma Pigmentosum, Cockayne Syndrome and Trichothiodystrophy. DNA Repair.

[5] Cleaver J.E. (1968). Defective Repair Replication of DNA in Xeroderma Pigmentosum. Nature.

[6] Setlow R.B., Regan J.D., German J., Carrier W.L. (1969). Evidence That Xeroderma Pigmentosum Cells Do Not Perform the First Step in the Repair of Ultraviolet Damage to Their DNA. Proc. Natl. Acad. Sci. USA.

[7] Epstein J.H., Fukuyama K., Reed W.B., Epstein W.L. (1970). Defect in DNA Synthesis in Skin of Patients with Xeroderma Pigmentosum Demonstrated In Vivo. Science.

[8] Paszkowska-Szczur K., Scott R.J., Serrano-Fernandez P., Mirecka A., Gapska P., Górski B., Cybulski C., Maleszka R., Sulikowski M., Nagay L. (2013). *Xeroderma pigmentosum* Genes and Melanoma Risk. Int. J. Cancer.

[9] Wade M.H., Plotnick H. (1985). Xeroderma Pigmentosum and Squamous Cell Carcinoma of the Tongue. J. Am. Acad. Dermatol..

[10] Chambon F., Osdoit S., Bagny K., Moro A., Nguyen J., Réguerre Y. (2018). Dramatic Response to Nivolumab in Xeroderma Pigmentosum Skin Tumor. Pediatr. Blood Cancer.

[11] Pardoll D.M. (2012). The Blockade of Immune Checkpoints in Cancer Immunotherapy. Nat. Rev. Cancer.

[12] Overwijk W.W., Wang E., Marincola F.M., Rammensee H.-G., Restifo N.P., for the Organizing Committee of the 2013 SITC Workshop on Personalized Immunotherapy (2013). Mining the Mutanome: Developing Highly Personalized Immunotherapies Based on Mutational Analysis of Tumors. J. ImmunoTherapy Cancer.

[13] Van Allen E.M., Miao D., Schilling B., Shukla S.A., Blank C., Zimmer L., Sucker A., Hillen U., Geukes Foppen M.H., Goldinger S.M. (2015). Genomic Correlates of Response to CTLA-4 Blockade in Metastatic Melanoma. Science.

[14] Anagnostou V., Bardelli A., Chan T.A., Turajlic S. (2022). The Status of Tumor Mutational Burden and Immunotherapy. Nat. Cancer.

[15] Kay J., Thadhani E., Samson L., Engelward B. (2019). Inflammation-Induced DNA Damage, Mutations and Cancer. DNA Repair.

[16] Zalaquett N.G., Kreidieh L., Youssef B., Mourad M., Kreidieh F. (2024). Case Report: Neoadjuvant-Intent Pembrolizumab Resulted in Complete Response in a Xeroderma Pigmentosum Patient with Locally Advanced Resectable Cutaneous Squamous Cell Carcinoma of the Nose. Front. Med..

[17] Kaste S.C. (2019). Imaging of Pediatric Cutaneous Melanoma. Pediatr. Radiol..

[18] Filippi L., Bianconi F., Schillaci O., Spanu A., Palumbo B. (2022). The Role and Potential of 18F-FDG PET/CT in Malignant Melanoma: Prognostication, Monitoring Response to Targeted and Immunotherapy, and Radiomics. Diagnostics.

[19] Sachpekidis C., Weru V., Kopp-Schneider A., Hassel J.C., Dimitrakopoulou-Strauss A. (2023). The Prognostic Value of [18F]FDG PET/CT Based Response Monitoring in Metastatic Melanoma Patients Undergoing Immunotherapy: Comparison of Different Metabolic Criteria. Eur. J. Nucl. Med. Mol. Imaging.

[20] Anderson T.M., Chang B.H., Huang A.C., Xu X., Yoon D., Shang C.G., Mick R., Schubert E., McGettigan S., Kreider K. (2024). FDG PET/CT Imaging 1 Week after a Single Dose of Pembrolizumab Predicts Treatment Response in Patients with Advanced Melanoma. Clin. Cancer Res..

[21] Iravani A., Wallace R., Lo S.N., Galligan A., Weppler A.M., Hicks R.J., Sandhu S. (2023). FDG PET/CT Prognostic Markers in Patients with Advanced Melanoma Treated with Ipilimumab and Nivolumab. Radiology.

